# Perspectives of family medicine residents on artificial intelligence for survival estimation in patients with serious illness

**DOI:** 10.1371/journal.pdig.0000917

**Published:** 2025-07-01

**Authors:** Gemma Postill, Anglin Dent, Jill Dombroski, Amol A. Verma, Jeff Myers, Tavis Apramian

**Affiliations:** 1 Institute of Health Policy, Management and Evaluation, Dalla Lana School of Public Health, University of Toronto, Toronto, Ontario, Canada; 2 Temerty Faculty of Medicine, University of Toronto, Toronto, Canada; 3 Li Ka Shing Knowledge Institute, St. Michael’s Hospital, Unity Health Toronto, Toronto, Canada; 4 Department of Laboratory Medicine and Pathobiology, University of Toronto, Toronto, Canada; 5 Division of Palliative Care, Department of Family & Community Medicine, University of Toronto, Toronto, Canada; Shahid Beheshti University of Medical Sciences School of Dentistry, IRAN, ISLAMIC REPUBLIC OF

## Abstract

As technology for artificial intelligence (AI) in medicine has rapidly proliferated, research is needed on *how* AI should be used in healthcare. Family physicians could deploy AI to predict survival in serious illness which is a particularly difficult task given the breadth of diseases encountered in primary care. Little research exists to inform whether survival estimation tools are welcome in primary care to manage serious illness prognostication. To address this gap, we elicited the perspectives of family medicine residents on the potential use of AI to help them predict survival (i.e., time expected) for their patients with serious illness. Our qualitative study draws on semi-structured interview data from 18 family medicine residents in Canada. We used a pragmatic framework to conduct our analysis, employing principles of constructivist grounded theory. We identified that family medicine residents were receptive to AI survival estimation for serious illness management, particularly for supporting their delivery of expert advice over a broad range of clinical topics. However, caring for patients with serious illness in primary care involves more than survival estimation, with such a tool having likely only limited applicability to end of life. Summarizing these perspectives, we identified four themes: (1) improving patient care with AI, (2) AI with a grain of salt, (3) patient-driven use of AI, and (4) augmenting, not replacing family physicians. Thus, survival estimation with AI for serious illness has potential clinical value in primary care. In addition to survival, pertinent challenges to address with AI include understanding of expected function, maximizing quality of life, and response to interventions, in addition to quantifying survival time. Future prognostication models should consider use of additional patient-centered outcomes and modifying the outcomes predicted based on prediction timepoints. To successfully deploy these technologies in primary care, additional education and role modelling of technology use is needed.

## Introduction

Most research on uses of artificial intelligence (AI) in medicine has asked “what *can* AI be used for” rather than “what *should* AI be used for?” [1] Use-cases for AI should be based on current clinical challenges for which physicians value AI support [2–4]. A thorough evaluation of such clinical challenges and their hypothetical solutions, prior to the development of solutions, is a key pillar of *design thinking* (e.g., a problem solving approach that focuses on solutions) and *human-centric design* (e.g., problem solving technique that puts people – users, observes, and those impacted – at the center) and can facilitate successful implementation of AI tools in healthcare settings [5,6].

Prediction of survival duration (e.g., survival estimation) for patients with serious illness (e.g., incurable and progressive medical conditions) through the automated synthesis of individual patient health information is one AI use-case [7]. AI-derived survival estimates could address prognostic uncertainty, a known challenge for family physicians aiming to adequately prepare their patients for progressive illnesses [8,9]. However, prior evaluation of family physicians’ perspectives on the applications of AI in their practice has largely assessed opinions of AI in general [10]. Little research exits to inform whether survival estimation tools would be welcome in family medicine.

Engaging clinical end users *prior* to the deployment of AI tools can improve their clinical relevance, acceptability, and safety [11–13]. Newly licenced physicians (e.g., medical residents) are the ultimate end users of the novel AI tools currently transforming the landscape of healthcare. Evaluation of their perspectives can also identify, and potentially address, concerns for automation bias [14–16]. Thus, our objective was to elicit the perceived utility and concerns of family medicine residents regarding a hypothetical AI tool for survival estimation among patients with serious illness. Such approach identifies contextualized opportunities, barriers, and risks of AI for illness prognostication in the primary care setting.

## Methods

### Study design

This qualitative study draws on semi-structured interview data from 18 family medicine residents (i.e., post-graduate medical trainees specializing in family medicines) in Canada. Interviews were conducted in a two-step process. Our second round of interviews allowed further probing of initial insights that arose in the first round in follow-up interviews [17].

### Participant recruitment

We invited current family medicine residents through their program’s mailing list to discuss how physicians prepare patients with serious illness for declining health. All residents who responded to the call for research were included in the study. Recruitment for the first round was ongoing as the initial interviews were conducted. For the second round, participants were recruited through email follow-up of those who completed the first round.

### Data collection

We completed interviews through *Zoom*, a teleconferencing software (University of Toronto Ethics Board #44805). Previous research has shown virtual interviews to be of similar quality to in-person interviews and often preferred by interviewees [18,19]. The interviewer’s (JD, TA, or GP) camera remained on for transparency and rapport [20].

Semi-structured interview guides were used to facilitate the conversation (S1 File). First round interviews (n = 18, conducted November 2023 to May 2024) lasted 45–60 minutes (mean: 52 minutes). The initial interviews focused on serious illness communication and the use of AI survival estimation tools. We aligned participants on a common definition of serious illness: any incurable or progressive illness, such as organ failure or metastatic cancer. Discussions on AI began with an open-ended question about a hypothetical AI tool to accurately predict how many weeks a patient had left to live. We invited all participants to a follow-up interview focused on alternative roles for AI in the care of serious illness. Second round interviews (n = 4, conducted August 2024) lasted 20–32 minutes (mean: 26 minutes).

All interviews were audio-recorded and transcribed verbatim by a member of the study team or professional transcriptionist. The transcripts were then checked for consistency with the audio file. Interviewing, transcription, and initial analysis occurred concurrently, and we ceased data collection upon reaching data saturation. The research team’s thought processes and analytic notes were documented via completion of memos throughout the study [21].

### Data analysis

We used a pragmatic framework to conduct our analysis, drawing on the principles of constructivist grounded theory [22] and inductively analyzing all transcripts [23–25]. We provide further detail on our positionality as authors and the theory that underpins our methodological approach in S2 File.

Data were coded to highlight insights and patterns in participants’ perspectives. Coding was done in duplicate by GP and AD with *NVivo* software (version 14) [26] after initial thought calibration following completion of the first five transcripts. GP and AD compared coded transcripts for consistency in coding, discussing with the larger research groups where discrepancies existed. Development of the initial codebook was done iteratively by GP and AD following coding through collaborative working sessions, in which their codes were triangulated to reach a consensus structure. Validation of the final codebook (S3 File) involved the larger research team and consisted of peer feedback through reviewing coded transcripts and discussing the alignment of the themes with associated codes. Disagreements about codes or themes were again resolved through consensus discussions. Meeting notes were recorded to establish an audit trail [27]. Where possible, *NVivo* labels were used (e.g*., grain of salt*) to retain participants original thoughts [22].

### Data reporting

We report only the parent codes with an exemplary quote to remain concise, with the full codebook available in S3 File. All quotes are presented with a unique and randomly generated study participant identification code. Reporting followed the Standards for Reporting Qualitative Research guidelines (S4 File) [28]. For reproducibility, the study protocol is available in S5 File.

## Results

Overall, participants (N = 18) were receptive to integrating AI in the management of serious illness, though identified some limitations of using AI for estimating survival in serious illness. Summarizing these perspectives, we identified four themes: (1) improving patient care with AI, (2) AI with a grain of salt, (3) patient-driven use of AI, and (4) augmenting, not replacing family physicians. Across the four themes, we constructed 17 parent codes and 55 child codes (S3 File).

### Theme 1: Improving patient care with AI

Participants believed AI survival estimation could potentially address challenges in the care of patients with serious illness in primary care, given the breadth in family medicine and the clinical value of having an accurate survival estimate (Box 1). Participants described family medicine to be “*miles wide and an inch deep*,” (**EE10S)** reflecting variability in the type of serious condition, stage of illness, and values of the patient (initial reaction, how much information they wanted, their goals of care, etc.). Even with such breadth, participants reported needs to synthesize and relay information at an expert level to support patient illness understanding. As a result, many *“always feel I’m lacking information. I think, specifically, when people ask about prognosis*…*it’s very, very tough unless you are very subspecialized in the condition that they have.”* (**AU16D)**

Box 1 **Parent codes, definitions, and exemplary quotes on *Improving Patient Care with AI* (Theme 1)**

**Prognosticating is challenging**. Participants describe inherent difficulty in prognosticating serious illness in family medicine due to its complexity and heterogeneity; they in turn report apprehension when approaching conversations about serious illness due to lack of confidence in their prognostication. AI prognostication of survival duration presents an opportunity to improve confidence and understanding of their patients’ trajectories.

*“There’s the classic question when someone asks how long they have, and that I feel is so patient dependent. I know even for some people that know someone’s case very well, it can still be really difficult to answer that question. But when I feel I also don’t know the information* […] *then I really struggle to give them a number, and I shy away from saying anything.” –* ***AE1K***

**Importance of accurate survival estimation in serious illness**. Participants emphasize the role of survival estimation in guiding treatment decisions, actioning goals of care, and maximizing quality of life. A common challenge is that patients and their families often do not understand the incurable and progressive nature of serious illness.

*“Yeah, I do think that just having that certainty that quote/unquote ‘deadline,’ I think too it probably would help in terms of making sure that we’re all in the same place – me, the patient, and the family. Because we’re all accepting that yeah, this is the week it’s going to happen* [patient’s death] *so this is how we would like to prepare for that. A lot of times I find when we’re not able to have those conversations, it is because the family, the patient, and the team are just not on the same page. Someone is in denial, whether it be the patient or the family.” –* ***AS17M***

**Managing expectations.** Participants explain that an AI survival estimation tool would provide a concrete starting point to initiate discussions of serious illness with patients, grounding patients and their family on a concrete timeline and thus, enabling more fruitful discussions of next steps.

*“*[Accurate AI survival estimation] *It is a good thing, honestly, because, again, you remember, my main concern was uncertainty sometimes. But now, if I am certain that this will happen at this time, I would give clear information to the patient. We will set clear expectations, and we will set clear goals of care, then the patient would be directed to the appropriate care and will have the appropriate plan that matches their wishes, their family wishes,* [and] *their future plans.” –* ***ER7R***

**Actioning goals of care.** Participants describe an AI survival estimation tool as supporting earlier and more productive surrounding goals of care discussions by allowing for personalized planning based on concrete timelines.

*“Let’s say AI tells us that this patient has a condition, that he has got only 6 months left to live. Then we would be changing into palliative care, talking about the patient’s wishes. Asking him to spend time with his family, trying to help him understand how things will be going into more comfort care, planning again with his consent, and having those discussions with him and his family, rather than going for an aggressive mode of treatment.” –* ***AN2F***

**Enabled efficiency with clinical decision support tools.** Participants discuss the utility of an AI survival estimation as akin to traditional clinical decision support tools (e.g., risk prediction calculators), which help synthesize information into an output. They also note that an added benefit with AI can be automated information synthesis if it interfaced directly with EMRs.

*“AI just speeds up the information gathering.” –* ***RI14B***

Effective communication of survival expectations to patients and their families was identified as a challenge and critical component of caring for patients with serious illness. Participants described their patients with serious illness often have a limited understanding of their prognosis. Participants proposed that AI survival estimation could help them to “*be more confident in asking those questions and navigating through the process* [of conversations around serious illness].” (**AN2F**). Participants tethered to the notion that accurate and definitive prediction of survival would eliminate some complexity in such serious illness conversations:

*“I do think that* [AI survival estimation] *could help because just having that certainty, that ‘deadline’, I think would help in terms of making sure that we’re all on the same page – me, the patient, and the family. We’re all accepting that this is the week it’s going to happen, so this is how we would like to prepare for that.” –* **AS17M**

AI has the potential to resolve ambiguity in survival estimation, which they positioned as a significant barrier to engaging in early support for patients with progressive disease.

### Theme 2: AI with a grain of salt

Participants considered the benefits of AI with a “*grain of salt*”, acknowledging its inherent risks and inappropriate use cases (Box 2). First, participants felt their lack of formal education in AI methodology and clinical use would impede their ability to safely and effectively leverage its benefits. Participants generally lacked an understanding of what AI is and how it works, often assuming all AI to be “*like ChatGPT.*” Participants commonly confused and equated generative (i.e., producing new content) and non-generative (i.e., forecasting clinical outcomes based on patterns in data) AI. Some referenced a specific need for structured learning, such as “*case-based learning on* [AI]*, sort of like a simulation… and having somebody to talk to in case there are more questions as people start to use* [AI tools].” (**UZ11J**)

Participants also expressed concerns for patient privacy and the potential for error, observing that AI survival estimation cannot always be correct.

*“I don’t feel like robots or machines can really work 100% accurately. I do believe in miracles. I do believe that things can happen that even doctors don’t believe is going to happen… So, it will almost be like a study vs. a machine. We can only, you know, take what it says with a grain of salt.” –* **IY1K**

In this sense, participants interpreted AI to be analogous to other tools in medicine, emphasizing the need to give patients the predicted outcomes with an acknowledgement of the potential for error. Interestingly, participants also questioned the accuracy of AI given the potential for human factors (treatment adherence, patients preferences, etc.) and divine factors (e.g., miracles) to change patients’ outcome after prediction, even if the model was correct at the time of prediction.

Box 2 **Parent codes, their definitions, and exemplary quotes on *AI with a grain of salt* (Theme 2)**

**Education to address lack of AI understanding**. In general, participants lacked a formal understanding of what AI is and how it works, often assuming all AI to be “*like ChatGPT*.” Participants described discomfort at the idea of justifying how AI operates to patients and expressed need for training on what AI is and how it works.

“*I definitely need a lot more training on how* [AI] *comes up with the factors that go into its decisions …* [I would need] *general training on what AI is so* [I] *could explain it to patients, and they could understand.”* – **EE10S**

**Concerns for new technology being wrong.** Participants note that AI’s prediction can be wrong, owing to modelling errors or their patient being exceptions to health trends captured in the model’s training data.

***“****And it’s a difficult thing, to get comfortable having those types of conversations with people and saying, ‘I’m confident that you’re not* [going to get better],*’ because nobody wants to be wrong, right? And I think sometimes we, even in medicine, we can think to some of those TV or movies, the media that we see where there’s miracle recoveries from things.”* – **OY3J**

**Human clinical gestalt is important for survival estimation**. Participants describe that current approaches to estimating survival involve incorporating features that cannot be quantified and that are based on clinician instincts and human factors.

*“I think it’s always a gestalt thing when you’re looking at the patient… I think thinks like frailty, weight, work of breathing… It’s very gut feeling based most of the time.”* – **EE10S**

**Need to ensure privacy with use of patient data**. Participants emphasize concerns for patient privacy with the integration of an external tool that generates predictions based on personalized patient data. Participants discuss the need to ensure patient consent prior to an external model accessing patients’ data. Participants noted that this may change with social license around secure data sharing and comfort in privacy measures.

*“I think it’d be a great tool, with some considerations for privacy and consent. It would help guide discussions with patients, but also sort of be used cautiously… Discussing with patients about this tool whether they want to use it and have their information be put into this tool.” –* **LS4A**

### Theme 3: Patient-driven use of AI for prognostication conversations

In discussing AI survival estimation, all participants emphasized the need to ensure patient-driven design and use of AI (Box 3). For example, participants believed that it *“has to be the patient’s choice if they wanted to know* [how much time is left].” (**BD6A**) Some participants expressed concerns that patients could be harmed by the knowledge that they have limited time, especially early in the course of the illness. Moreover, many participants believed such a tool would only be useful in the patient’s final weeks of life, as patients (and families/caregivers) decide how to balance quantity and quality of life.

*“If it was someone who was more on a more acute trajectory, then it would be a little bit more of a serious conversation around, you know, let’s make sure we’re making the best decisions now and making sure everyone is on the same page, versus being able to take a little bit more of a wait-and-see approach, and waiting for the family and patient to come towards you and meet you halfway.” –* **EE10S**

Patients with serious illnesses have evolving needs and the role of family physicians is addressing the current needs of the patient at each visit. Therefore, an AI survival estimation tool would not be intended for use in all visits. Indeed, one participant (**UZ11J**) described care of their patient with serious illness saying, *“I need to cover* [their] *needs.* […] *we need to go day by day, I guess. The most important thing is what’s happening right now, not in the future, if that makes sense.”* Thus, while predicting the amount of time left can be useful in some instances, models predicting other patient-centered outcomes (e.g., symptom burden, quality of life, and treatment efficacies) may be leveraged more frequently throughout the trajectory of serious illness care in family medicine.

Box 3 **Parent codes, their definitions, and exemplary quotes on *patient-driven use of AI for prognostication conversations* (Theme 3)**

**Correct use case of patients who would benefit.** Participants stress the need to clarify the utility of AI-based prognostication, identifying the correct populations, illnesses, and timepoints within illness trajectory for which the tool can be used. Participants note that patients and their family typically most value information about survival duration at diagnosis and in their final weeks and months of life.

*“But in terms of your more standard life-limiting illness, like the longer courses of cancer* […] *two, three years out. So we’re having those conversations over time and adjusting appropriately. So, I think for those,* [accurate surviving estimation] *wouldn’t make a difference, but yeah, certainly, obviously, for sudden illnesses, sudden traumatic deaths and stuff like that,* [an AI prognosis tool] *would obviously change how we’d approach those patients.” –* **EE10S**

**Concerns for whether patients can handle accurate and specific prognosis**. Participants have reservations regarding the benefits of an AI prognostication tool across all patient situations, with concern that some patients could be harmed by the knowledge of their specific life expectancy .

*“In my mind, I just think it can do more harm than good, because you want the time that you have to be spent with family and all. But in the back of your head, if there is like a timeline and there is like a ticking clock that it’s the end of the week or two weeks, whatever, it just ruins the whole experience, or it can really make it darker.” –* **BD13A**

**Paternalism and Patient Autonomy**. Participants describe being conflicted when evaluating whether the patient needs to know how much time is left, often worrying that they will do more harm than good. Participants frame this as an ethical decision.

*“Is it ethical for me to be relaying this information? Am I doing more harm than good by relaying this information or not?”* – **AA18S**

**Patient-centered prediction outcomes are of greater value than survival estimation**. Participants note that while survival estimation can be useful in some instances, prediction of more patient-centered outcomes (e.g., symptom burden, quality of life, and treatment efficacies) may be of greater utility in the care of their patients.

*“*[Outcomes of greater value include] *odds of having palliative symptoms – like having severe pain in the future, severe constipation, shortness of breath – just so they are prepared to deal with this symptom that people have at the end of their life. If somebody probably is more likely to have pain, they are then very well prepared to address it. Maybe even in advance, have some interventions, even psychological, to be prepared. Things like that*.” – **UZ11J**

### Theme 4: Augmenting, not replacing family physicians

Participants believed AI could augment care decisions but noted that AI survival estimation could not replace nor comprehensively address the role of the family physician in caring for those with serious illness (Box 4). One participant noted:

***“****The prognosis sometimes is certainly not everything. There’s more to that discussion and how you can support your patients than getting them ready with that* [survival estimation] *information. There’s a lot more that goes into the support of the patient and how they make the decisions and things.****” –* AY5M**

Residents understood the scope of family medicine in the case of caring for patients with serious illness to be broad including providing longitudinal care, emotional support, illness education, and referrals to resources. Owing to this, **ER7R** said, *“The best person to talk to their patients about their illness, their prognosis, is their family doctor. They know the psychosocial aspect of the patient’s life, the patient’s expectations, the patient’s wishes, all that stuff. I think it is a crucial, very important role.”* Thus, while AI survival estimation can be useful, the role for family physicians in caring for those with serious illness was believed to be impossible and unwise to attempt to replace with an algorithm.

While AI automation of survival estimation was viewed as a potentially valuable tool, residents were clear it would need to be more accurate than physicians, as well as improve clinical efficiency and patient outcomes, to truly be beneficial. Participants believe that not all physicians will experience the same gains in efficiency from each AI tool; rather, its value would vary physician-to-physician and patient-to-patient, with it being of greater value for junior trainees and particularly challenging patients.

Box 4 **Parent codes, their definitions, and exemplary quotes on *Augmenting, not replacing family physicians* (Theme 4)**

**Longitudinal therapeutic relationship is the core of family medicine**. Participants acknowledge their role as family physicians in the management of serious illness to involve developing trust, building rapport, and providing emotional support to patients.

*“I’m providing holistic care, I’m taking in consideration every single aspect that could be related to the patient’s health, and I think this is the best approach that we can do, because there’s no definitive treatment for everything. There’s no best medical care approach. So, I think involving the patient in their care and letting them take the lead would help us also to make the best decisions.” –* **ER7R**

**Physicians with AI will replace those without AI**. Participants highlight that while survival estimation is part of care of serious illness, there is substantially more involved, including providing emotional support and connecting patients to the necessary resources. Even knowing patients’ prognosis more accurately, their roles as a family physician would not change; they would continue to address patients’ current needs.

*“Because me as a physician, I can know [the AI survival estimation] and I don’t think that will change my practice. I want to be there for the patient, covering all their needs or supporting the patient as much as they want, even though I know the date where the patient will die.” –* **UZ11J**

**Efficiency looks different physician-to-physician**. Participants describe specific areas within their practice where AI can or has previously increased efficiency. Participants note that all physicians will not experience the same gains in efficiency or accuracy from each AI tool.

***“****Our clinic just rolled out an AI scribe where you just press a button. It listens to the conversation. It makes a note. And it’s this incredible thing for the old school doctors who can’t type and talk at the same time. It’s a godsend. I tried it. I didn’t really like it. I felt the note that it generated was very clunky and didn’t look very good, not very organized. I liked my way of doing it because I use a template. And then I just type while I talk and fill out the template. But I can see how somebody who cannot type and talk, who has to draft up a note after the interaction is gone and patient is out the door, how an AI would be really good. Super helpful*.” – **RI14B**

**Physicians to guide introduction of AI in clinical care**. Participants reference the salience of physician leaders in AI implementation so that there is consideration to the patient perspective and trainee perspective, alongside that of institutional policy makers and established clinicians.

*“People should be encouraged to use it and to experiment with it. Veteran users should demonstrate how good it is and encourage new learners to try it out. There should be as few barriers as possible. It should be made easy for new docs to try it out.”* – **RI14B**

## Discussion

Using principles of design thinking, [29,30] we evaluated the perceived clinical utility of a hypothetical solution (AI-derived survival estimates) to support the care of patients with serious illness prior to the development and implementation of such solutions. Family medicine residents believed AI-derived survival estimates could support patients with serious illness, though they identified pertinent risks and challenges. Together, these thoughts are summarized by four themes around AI survival estimation: (1) improving patient care, (2) grain of salt, (3) patient-driven use, and (4) augmenting, not replacing family physicians. Our findings highlight how and when AI survival estimation can augment prognosis conversations in primary care, and can thus, guide development of AI survival estimation tools.

Family physicians are known to be largely positive about AI, with attitudes hinging on the context of adoption [31]. Perceptions of AI are more favorable for automating administrative tasks than for making clinical decisions [32]. We extend on prior general knowledge by exploring in-depth perspectives on a specific use-case and clinical context for AI in primary care. With our approach, we identify the following considerations for developing and deploying AI models for survival estimation of serious illness in family medicine:

***AI derived survival estimation would be valuable in certain contexts and for certain patients*.** Participants believed having accurate survival estimation is clinically important as it provides an objective starting point for prognosis conversations, potentially improving patients’ illness understanding at diagnosis and the alignment of care with patient goals. Indeed, participants noted how survival estimation may be most important in initial serious illness discussions (e.g., diagnosis), throughout management (e.g., when new complications arise), and in the final weeks of life. The implication for model development is that such models should not run automatically but be triggered by clinicians when appropriate in a patients care. Such recommendation also aligns with environmental consideration for AI and with participants’ views that AI derived survival estimation is most valuable for patients where prognostication support is required (e.g., difficult to anticipate clinical trajectories). Likewise, such a model would likely be of use if its design facility access to both specialists and family physicians, enabling their collaborative management of patients with serious illness.

***Survival estimation has ethical implications and use of such tool must be patient- and family-oriented.*** Patients may not want to know the time they have left. Survival estimation would also need to be highly accurate as it may have serious impacts on care choices; in such clinical scenarios, the use of AI as *augmented intelligence* (e.g., integrating AI with clinical insights) is likely the most appropriate route. Considerations of liability in the context of augmented prognostication need to be considered thoughtfully, particularly in instances of shared liability with developers or hospitals. However, participants of our study proposed that survival estimation could theoretically alter the outcome, and thus, were concerned that accuracy, performance bias, and dataset shift over time of such a model could never truly be accurately assessed. To address these concerns, predicting ranges of time alive (vs. exact number of weeks) and prompting physicians to inquire whether patients desire the information before communicating it with patients may ensure the model is more clinically meaningful.

***Prognostications of serious illness involves more than the number of weeks left to live.*** Care for patients with serious illness involves ensuring illness understanding, aligning care plans with patient goals, addressing patients’ current symptoms, and liaising needed psychosocial supports. Indeed, having a numeric survival estimate in serious illness communication is much less important than family physicians believe [33]. Palliative care research suggests patients value time alive *and at home* more than time alive [34]. Ensuring relevant and clinically meaningful outcomes will positively influence the value of an AI tool and its perception as complementing (vs. replacing) clinical judgement [35]. To address these concerns, AI prediction tools could instead integrate multiple patient-centered outcomes (e.g., predict response to therapy, functional outcomes, or complications) on one model interface, or alternatively be used to improve patient’s illness understanding (i.e., as a chatbot or to generate tailored descriptions of expected trajectories).

***Successful AI deployment requires education and role modelling*** By elucidating perspectives on an AI tool with a specific use-case (i.e., survival duration prediction), we identified AI educational gaps needed prior to implementation. Lack of formalized education and familiarity has been reported as a barrier to AI acceptance in family medicine [35]. Family medicine residents were not confident in their understanding of AI and confused generative and non-generative AI. Understanding these concepts will be critical for safely leveraging AI in clinical care as they allow physicians to correctly identify relevant limitations such as algorithmic biases and hallucinations. Further, participants desired role modelling of integrating novel AI technologies into clinical workflow. These findings support the Diffusion Innovation Theory, which outlines the trajectory of achieving widespread acceptance of a novel idea within an established population or social system. Our findings specifically highlight *trialability* and *observability* for successful AI deployment [36]. Overcoming well-described deployment barriers [37] should also involve strategies to highlight perspectives of early AI adopters within primary care.

To date, no general AI mortality or prognosis estimation models have been approved or deployed in primary care in Canada. Prior AI survival estimation models have shown strong performance but have predominantly been designed for oncology, [38] and are disease specific – such as for lung cancer, [39] skin cancer, [40] and breast cancer [41]. These models have primarily focused on prediction of survival at fixed time points (i.e., 2-year mortality), with few models estimating survival time directly. We were interested in exploring the use of a general prognostic model by generalist physicians, analogous to the use of the Palliative Performance Scale [42] or other general prognostic tools. This is particularly compelling to study because the information gained from a prognostic model might be more informative to a generalist clinician who has less expert knowledge of the anticipated trajectory of a given illness. Further, taking a hypothetical non-disease-specific model, allowed us to explore general concepts about the use of such models that are applicable to disease-specific and non-disease-specific models alike, whereas anchoring our study to a specific prognostic model would have narrowed the applicability of our findings to the specific strengths and weaknesses of that model.

Another consideration – and source of diversity across prognostication models – is how patients’ medical information is extracted and inputted into the model. Many require physicians to extract the medically relevant information. For example, an oncology survival estimation model leveraged the unstructured text on a doctor’s note to predict survival [43]. Indeed, AI model performance is closely linked to data (quality, comprehensiveness, format) provided [44]. Such reliance on data structure and quality is one reason why the performance of an AI model will often drops when deployed in a new clinical setting [45]. As noted by our participants, models that do not automatically extract the clinically relevant information from electronic health records have limited value add, as challenges in prognosticating are identifying, documenting, and synthesizing all medically relevant information.

Integrating principles of human-centric (or solutions-oriented) design and eliciting stakeholder perspectives prior to prototyping can facilitate successful implementation and impact in healthcare settings. A salient clinical example of such an approach is PODS (Patient Oriented Discharge Summaries) – the development of which involved stakeholder feedback prior to its design, implementation, and evaluation [5,6]. Indeed, this type of approach is largely missing from the medical AI landscape, with most work to date focusing on what AI *can* do, versus what AI *should* do. For such reason, we identified a clinical challenge and evaluated the clinical utility of hypothetical AI solutions, identifying considerations for its clinical acceptability and utility.

## Strengths and limitations

Strengths of our study include the use of a flexible semi-structured interview approach and two interviewing rounds, which enabled participants to guide the discussion and the research team to follow up on ideas not anticipated in the first round. Regarding its limitations, our exploratory research included only a subset of family medicine residents in Canada with a sample size of 18. We did not collect sociodemographic data of these respondents. However, the purpose of the study, built from a constructivist paradigm, was to describe meaningful stories of conceptual interactions between AI and mortality that might resonate with researcher and policy-makers to begin a new scholarly conversation [46]. Although our findings might be conceptually transferable (i.e., they apply to the experiences of individuals outside of our sample), they are not intended to be statistically generalizable [47]. Our findings may not reflect all physicians currently established in their practice owing to generational differences in physician training and comfort with AI prognostication. To ensure we provided meaningful and useful results that might be transferable to similar contexts, we used Nowell’s criteria of trustworthiness (credibility, transferability, dependability, and confirmability) [20,21,48,49]. Additionally, our findings provide the perspectives of family medicine residents prior to the deployment of AI survival estimation, and thus, may not be reflective of those post-deployment. Future research should evaluate the perspectives of all individuals potentially affected by such tool (e.g., patients, bioethicists, and hospital decision makers) prior to deployment as well as when such tools have been clinically deployed.

## Conclusion

Our qualitative study identified that family medicine residents were receptive to AI-derived survival estimates for patients with serious illness, particularly given the need to deliver expert advice over a broad range of disease states in primary care. However, AI tools were viewed as supporting tools to achieve improved accuracy with survival estimation, rather than as providing definite survival estimation calculations, particularly given the need to integrate elements of the clinical gestalt, visual and sensory information (e.g., patients’ work of breathing), and patients’ goals of care. Additionally, caring for serious illnesses involves more than survival estimation. Pertinent clinical management challenges that may additionally be addressed with AI include building illness understanding and anticipating functional trajectories and quality of life outcomes. Future prognostication models for serious illness should consider use of patient-centered outcomes and varying model outcomes based on prediction timepoints and patient needs. To successfully deploy these technologies, clinical acceptance should be fostered through role modelling from early AI adopters.

## Supporting information

S1 File Interview Guide. (DOCX)

S2 File Positionality Statement. (DOCX)

S3 File Codebook. (DOCX)

S4 File Reporting Checklist. (DOCX)

S5 File Study Protocol. (DOCX)
