# Supplementary material for: Perspectives of family medicine residents on artificial intelligence for survival estimation in patients with serious illness
Source: PLOS Digit Health. 2025 Jul 1;4(7):e0000917. doi: 10.1371/journal.pdig.0000917 (PMC12212547; doi:10.1371/journal.pdig.0000917)
Supplement: S1 File — (DOCX) [file pdig.0000917.s001.docx]

**Semi-Structured Interview Guide – First Round Interviews**

1. How does your healthcare provider support you if you have advanced end-organ disease or metastatic cancer?
2. What kind of conversations have you received on this subject?
3. Have you had any conversations or explored any information regarding medical assistance in dying?
4. What do you know about medical assistance in dying?
5. What feelings do you have about your serious illness?
6. Do you worry that you may not understand prognosis?
7. Do you worry that discussing prognosis with your healthcare provider will be harmful?
8. Do you worry that discussing prognosis will lead to questions about medical assistance in dying?
9. What is the role of the family physicians in discussing prognosis?
10. What might it mean if your healthcare provider split the work of *preparing* patients with serious illness for worsening health from the work of making medical decisions?
11. If artificial intelligence was capable of accurately predicting prognosis, how would it change the healthcare providers interactions with you about your serious illness?
12. How does the electronic medical record and digital communication space shape the way you discuss serious illness with patients and colleagues?

**Semi-Structured Interview Guide – Follow-up Interviews**

*The interview will be recorded…*

*Today we are discussing artificial intelligence in the context of family medicine, specifically, in caring for serious illness. Your first interview was on serious illness in general. You covered several different components of the care of serious illness in the family medicine context – including MAID, EMRs, and AI. Today we will focus on AI*

- **Do you know what AI is? (e.g., can you define it?)**
- **What kinds of exposure have you had to AI?**
  - Clinically?

**Reminder of the interview with jill** If you will recall the last interview,

*So, the rest of my questions are going be around AI in serious illness in family medicine. When you spoke with Tavis. he gave you a hypothetical example of an AI tool that could predict how many weeks somebody had left to live and get that quite accurate. With the rest of the questions, we'll just keep that example in mind, because there's so many different uses of AI that I think it helps to be talking about the same use case. With a tool like that, how comfortable would you feel using it clinically.*

- **Is anticipating the how much time is left something that is challenging for you?**
  - Why or why not?
- **Would patients want to know this information?**
  - Why do you think patients don’t want to know?
- **How would you communicate this AI prognosis to patients and their family?**
  - How would you share this AI prognosis differently from how you normally share prognoses or bad news with those who have a serious illness?

- **What information would you need provided alongside the AI prognosis?**
  - (Examples: resources, explanation of why it is predicting, their treatment options, etc.)
- **Do you feel comfortable using AI risk prediction tools?**
  - Do you feel comfortable explaining AI prognoses?
- **Do you feel equally, more, or less comfortable using a personalized AI risk prediction tool compared to a typical statistical risk prediction tool (for example PPS score)**
  - Why?
  - What about AI risk prediction makes you uncomfortable?
- **Do you think you would need to explain to patients that  an AI model  was used to determine their prognosis?**
  - Why?
  - What about AI make you feel the need to have patient consent to use?
- **What ethical considerations do you need to consider with a tool like this?**

- - Would you inform patients that you are using AI? Why?
- **To use a tool like this, what type of education do you, as a physicians, need?**
  - *What* specifically do you need to know?
- **Instead of predicting the amount of time left, what would be more beneficial instead? To assist you in having these prognostication conversations?**
  - To manage patient reaction?
  - To provide emotional support?
  - To plan next steps / planning around quality of life
    - How do you currently identify these as a clinician?
- **Does the distinction between generative and predictive AI mean anything to you?** *[Explain if participant unsure]*
  - Would one be more useful?
  - Do you feel more comfortable using one, compared to the other?
- **Is there anything else that you would like to share with us?**

*That concludes our interview. Thank you for your time.*
