# Supplementary material for: Perspectives of family medicine residents on artificial intelligence for survival estimation in patients with serious illness
Source: PLOS Digit Health. 2025 Jul 1;4(7):e0000917. doi: 10.1371/journal.pdig.0000917 (PMC12212547; doi:10.1371/journal.pdig.0000917)
Supplement: S2 File — (DOCX) [file pdig.0000917.s002.docx]

**Positionality Statement**

**Theory Underpinning the Research Approach**

We used a pragmatic framework to conduct our analysis, drawing on the principles of constructivist grounded theory^1^ and inductively analyzing all transcripts. Constructivist grounded theory is used in health research to enable moral, ethical, and legal forms of social action to emerge from the analysis.^2–4^ We embed principles of constructivist grounded theory in a pragmatic approach for creating our codes and themes.^2–4^ We used such approach as our study was largely prospective, given there are no physical nor social processes by which artificial intelligence (AI), for survival estimation is used in family medicine. Similar approaches to anticipatory social research have shown success.^5^

**Research Team**

Our research team brings together a diverse range of expertise from AI development, clinical deployment of AI, qualitative research, medical education, palliative care, internal medicine, and family medicine, providing a comprehensive approach to studying AI's role in survival estimation among individuals with serious illnesses in the context of family medicine. Each member of the team contributes unique perspectives and skills, enriching the interdisciplinary nature of this project.

**Gemma Postill (MD/PhD student)** works at the intersection of AI and healthcare, with her research focusing on the use of AI for survival and functional outcome prediction. Her expertise in both qualitative research and AI equips her with the ability to critically analyze AI's applications, limitations, and ethical considerations in clinical decision-making. Gemma is also the education student co-lead of the Temerty Centre for Artificial Intelligence Research and Education in Medicine (T-CAIREM).

**Anglin Dent (MD/PhD student)** shares similar expertise in AI and qualitative research, with her research focused on prospective evaluation of AI deployment in clinical care. Anglin approaches AI in healthcare with a strong emphasis on deployment, focusing on how technology can complement humanistic approaches to care. Anglin is also a member of T-CAIREM.

**Jill Dombroski (PhD)** offers a specialized focus on serious illness research, end of life care, and qualitative methodologies. Her research centers on understanding the unique experiences and needs of these populations, especially in relation to end-of-life decision-making.

**Amol A. Verma (MD)** has substantial expertise in the development and clinical implementation of predictive AI models. Dr. Verma’s clinical experience combined with his technical knowledge positions him to critically assess the translation of AI models into real-world healthcare settings. His insights into how these models perform in practice help the team ensure that the tools developed are both clinically relevant and operationally feasible.

**Jeff Myers (MD)**, an expert in palliative care and serious illness communication, brings an essential focus on the compassionate and person-centered care required when dealing with serious illness at all stages.

**Tavis Apramian (MD, PhD)**, with his expertise in palliative care, family medicine, and medical education, provides a holistic understanding of the role AI can play in family medicine education. Such perspective allows understanding of the developmental zone of our participants and consideration of how AI tools align with the values of family medicine, such as fostering strong patient-provider relationships and ensuring equitable access to care.

Our research approach is interdisciplinary and grounded in the need to anticipate identify both the technical and human aspects of implementing AI in healthcare. Our collective aim is to explore how AI can augment, rather than disrupt, the compassionate care that is central to the treatment of individuals with serious illness in family medicine. We believe these findings can shape and encourage ethical, effective, and person-centered use of AI in medicine. We acknowledge the ethical, social, and practical complexities that arise when using AI in clinical settings and strive to address these in our work.

**References**

1. Charmaz, K. *Constructing Grounded Theory*. (Sage Publications, London ; Thousand Oaks, Calif, 2006).

2. Morgan, D. Pragmatism as a Basis for Grounded Theory. *Qual. Rep.* (2020) doi:10.46743/2160-3715/2020.3993.

3. Mohajan, D. & Mohajan, H. K. Constructivist Grounded Theory: A New Research Approach in Social Science. *Res. Adv. Educ.* **1**, 8–16 (2022).

4. Rieger, K. L. Discriminating among grounded theory approaches. *Nurs. Inq.* **26**, e12261 (2019).

5. Postill, G., Halpin, M., Zanin, C. & Ritter, C. ‘I’ve never been so stressed in my life’: a qualitative analysis of young adults’ lived experience amidst pandemic restrictions in Prince Edward Island, Canada. *BMJ Open* **14**, e075567 (2024).
