# Supplementary material for: Perspectives of family medicine residents on artificial intelligence for survival estimation in patients with serious illness
Source: PLOS Digit Health. 2025 Jul 1;4(7):e0000917. doi: 10.1371/journal.pdig.0000917 (PMC12212547; doi:10.1371/journal.pdig.0000917)
Supplement: S3 File — (DOCX) [file pdig.0000917.s003.docx]

**S3 File. Artificial Intelligence for the Prognosis of Serious Illness: A Qualitative Study of Family Medicine Resident Perspectives**

**Table A.** Codebook of Themes for Residents’ Perceptions of the Impact of AI-guided Prognosis on Management of Serious Illness

| **Parent Code** | **Child Code and Example** |
| --- | --- |
| **Theme 1: Improved patient care with AI survival estimation** | |
| **Prognosticating is challenging** | **Miles wide and an inch deep**: Participants emphasize the heterogeneity of serious illness in family medicine, with the need and expectation to know a little bit about a lot of illnesses (vs. depth on a subset of illnesses). Given the breadth of family medicine, participants feel it is challenging to relay the information patients need at an expert level.  “Family medicine is a mile wide and an inch deep type thing. Like, I don't know how to question some of the specialists. If they are, for a COPD patient, if they are on, you know, fourth-line treatment type things, there's not a lot I can add there, and hard for me to question decisions being made by the specialist in that sense.” – **EE10S**  “I find that I am often lacking the information or always feel I’m lacking information. I think, specifically, when people ask about prognosis […] it’s very, very tough unless you are very subspecialized in the condition that they have.” – **AU16D** |
|  | **Every patient is different**: Participants emphasize that even within a specific illness, every patient (and their families) will react differently to various styles of communication and information sharing, particularly in the context of serious illness. In that sense, there is no one-size-fits all for the level of knowledge patients want or the expected illness understanding from certain information.  “The reason why it [conversations with patients with serious illness] is hard is because each patient is so different. It’s like a whole universe. So, I don’t think there’s one thing that applies to all of them.” – **UZ11J** |
|  | **Challenging to prognosticate**: Participants describe inherent difficulty in prognosticating serious illness in family medicine due to its complexity and heterogeneity; they in turn report apprehension when approaching conversations about serious illness that they cannot confidently prognosticate. To them, AI-based survival estimation / prognostication presents an opportunity to improve confidence and understanding of their patients’ trajectories.  “There’s the classic question when someone asks how long they have, and that I feel is so patient dependent. I know even for some people that know someone’s case very well, it can still be really difficult to answer that question. But when I feel I also don’t know the information […] then I really struggle to give them a number, and I shy away from saying anything.” – **AE1K**  “I often don't know the prognosis of. I mean I can only speculate a lot of the times on what I guess is a close prognosis. Patients will often ask how long they have to live, how long before things change, or things get worse. And I can only estimate, and I always have to, you know, qualify my answers and things and then I think that would really change things. I think it [AI] would also change how I would approach that conversation, because some people may not want to know, at least with that level of certainty. So, you have to kind of have that discussion ahead of giving them that information. But yeah, it would be helpful, but also definitely would make the conversations different.” – **AY5M** |
|  | **AI reducing prognosis uncertainty**: Participants discuss how AI-based prognostication could improve residents’ ability to prognosticate their patients’ illness trajectories through reducing uncertainty around outcomes.  “I often feel a lot of uncertainty about […] how much time in terms of months, years, that kind of thing. And I feel like we often defer […] to the oncologist, specialist, or something like that. Just because we don’t always have that level of experience or knowledge to give specifics when it comes up.” – **AU16D** |
|  | **Knowing prognosis reduces provider distress**: Participants describe feeling distressed during, or avoiding altogether, conversations regarding serious illness when they feel lacking information.  “[If I couldn’t prognosticate] it would make it more unpredictable. […] It would also make it more emotionally distressing as a provider to not know, you know, how long people had, or you know, when to expect people to decline.” – **AY5M** |
|  | **Experiential learning**: Participants describe how most learning around prognostication (including survival estimation) and management of serious illness, particularly with respect to learning how to communicate with patients, occurs through practice and observing  “[I] had a couple of practice opportunities with standardized patients in medical school… but I think [now] primarily talking around real life cases in clinical settings is where a lot of learning how it [is done] and how these discussions affect people. Experiential learning is probably a better way to put it – a debrief after a family meeting or a difficult patient” – **AY5M** |
| **Importance of accurate prognosis in serious illnesses** | **Prognosis is central**: Participants emphasize that AI would provide the greatest impact on patient care by eliminating the uncertainty around prognosis. They discuss the role of prognosis in guiding treatment at all time points, influencing patient goals of care and making decisions that maximize quality of life.  “[Accurate AI prognosis] I think it would (help) because I would feel more equipped to answer their questions about their prognosis. I feel you would still have to be very patient-centered and ask them how much they want to know and understand their wishes for understanding their own prognosis. But I think I would feel personally better about being able to say, if they asked me, when it’s going to happen.” – **AE8K** |
|  | **Survival estimation more important with acute trajectories**: Participants emphasize the value of knowing how long a patient has to live, is of most benefit in patients’ final weeks of life, as patients decide how to balance quantity vs. quality of life and family members help to guide next steps.  “But if it was someone who was more, you know, on a more acute trajectory, then it would be a little bit more of a serious conversation around, you know, let’s make sure we’re making the best decisions now and making sure everyone is on the same page, versus being able to take a little bit more of a wait and see approach and waiting for the family, patient to come towards you and meet you halfway.” – **EE10S**  “But in terms of your more standard life-limiting illness, like the longer courses of cancer, I don't think it really changes that much because I'm coming from a place where there's that focus on the very early intervention on the palliative care side of things where we’re trying to get people two, three years out, so we’re having those conversations over time and adjusting appropriately. So, I think for those, it wouldn’t make a difference, but yeah, certainly, obviously, for sudden illnesses, sudden traumatic deaths and stuff like that, it would obviously change how we’d approach those patients.” – **AA18S** |
| **Managing Expectations with accurate prognosis** | **Setting clear expectations with AI enabled certainty**: Participants explain that an AI survival estimation could provide a concrete starting point to initiate discussions of serious illness with patients, allowing physicians to assess the patient’s illness perception and identify gaps to ensure clear expectations for the course of their illness.  “[Accurate AI prognosis] It is a good thing, honestly. because, again, you remember, my main concern was uncertainty sometimes. But now, if I am certain that this will happen at this time, I would give clear information to the patient. We will set clear expectations, and we will set clear goals of care, then the patient would be directed to the appropriate care and will have the appropriate plan that matches their wishes, their family wishes their future plans.” – **ER7R** |
|  | **Everyone on the same page with objective prognosis**: Participants refer to accurate knowledge of time left (prognosis) as something that can ground patients and their family on a concrete timeline, enabling more fruitful discussion of the next steps. In that way AI-prognosis helps ensure that patients, family members, and the entire medical team are on the same page.  “Yeah, I do think that just having that certainty that quote/unquote ‘deadline,’ I think too it probably would help in terms of making sure that we’re all on the same place, me, the patient, and the family. Because we’re all accepting that yeah, this is the week it’s going to happen [patient’s death] so this is how we would like to prepare for that. A lot of times I find when we’re not able to have those conversations, it is because the family, the patient, and the team are just not on the same page. Someone is in denial, whether it be the patient or the family.” – **AS17M**  “[If AI could help management of serious illness] Yes, I do think that could (help) because just having that certainty, that “deadline”, I think would help in terms of making sure that we’re all on the same page – me, the patient, and the family. We’re all accepting that this is the week it’s going to happen, so this is how we would like to prepare for that.” – **AS17M** |
| **Actioning goals of care with AI** | **AI to enable goals of** **care with timeline**: Participants emphasize that AI survival estimation would support an earlier, more productive, and/or more effective discussion surrounding goals of care in the management of serious illness and allow for personalized advanced care planning based on a concrete timeline.  “Let’s say AI tells us that this patient has a condition, that he has got only 6 months left to live. Then we would be more changing into a palliative care, more talking about the patient’s wishes. Asking him to spend time with his family, trying to help him understand how things will be going into more comfort care, planning again with his consent, and having those discussions with him and his family. Rather than going for an aggressive mode of treatment.” – **AN2F** |
|  | **Supporting personalized management, balancing quantity and quality of life**: Participants emphasize that AI timeline (personalized and accurate) would allow for care decisions to be based on whether life-prolonging care aligns with patients’ quality of life priorities.  “A lot of the challenges come down to how we can effectively care for them for both the quality and quantity of life point of view, knowing that it's often hard to do that work with one foot in each pathway. […] I think a lot of the difficulty comes down to being able to accurately prognosticate and help a patient understand where they are at in their disease trajectory, and […] what those goals are depending on where they are in that process.” – **OY3J**  “It would be helpful, as we would be managing and tailoring the treatment plans according to the timelines of the AI (prediction).” – **AN2F** |
|  | **Adding to risk vs. benefit decision**: Many participants describe insight gained from an AI prognostication support tool as potentially beneficial for making decisions around balancing aggressive treatment for serious illness with quality of life, which they identify to be challenging decisions.  “[Even with AI] it’s always like risk versus benefit. If there’s no benefit to a treatment plan anymore or the risks outweigh the benefits, then just aligning that with the patient’s best interests.” – **VY15E** |
| **Enabled efficiency with**  **clinical decision support tools** | **Efficient use of resources**: Participants discuss the utility of an AI survival estimation as guidance for decisions on when to pursue aggressive versus comfort interventions. Ultimately, they believe such a tool could improve resource management, as a more certain prognosis would likely lead to less aggressive treatment plans for some patients.  “I think that could save the resources, the burden on the doctors, the patient understanding […] all of these things. That could be helpful.” – **AN2F**  [In response to their perception of the potential of AI in family medicine] “We have busy practices. We see a lot of patients. Because we’re family physicians, we’re supposed to know everything, or at least respond to all the questions patients have… I use [AI] to speed up things and get specific, evidence-based answers.” – **UZ11J** |
|  | **Integrating multiple sources of information faster**: Participants emphasize that the value of AI-drive prognosis would be its ability to synthesize information faster than they can manually, drawing on relevant insights from multiple aspects of patients’ charts to create a final prognosis.  “AI just speeds up the information gathering.” – **RI14B** |
|  | **Technology already guiding prognosis**: Participants emphasize current utility of non-AI based prognostic calculators in care of patients with serious illness.  “There are a number of calculators that give prognosis factors that I may not have been familiar with and seeing them through the EMRs can better equip me to discuss something with a patient, you know. For example, if those calculators are a little more favorable.” – **LS4A**  “Yeah, I’ve seen their [EMR] notes. Seeing their test results as well is helpful. It’s hard to make decisions [on patient care] without an EMR” – **AY5M** |
|  | **AI interfacing with electronic medical records**: Participants emphasize how EMRs provides an interface to integrate patient data and provide a summary of the illness trajectory and understanding, and that AI could leverage the data from EMRs to create the templates needed to summarize patient illnesses and facilitate discussions of serious illness management and goals of care.  “I think it would be really helpful if we had the information at hand, because then we would know what questions we have to ask and not miss anything. We’d be more confident in asking those questions and navigating through the process. Especially with maybe a form or written template. That could be really helpful to make sure that we have not missed any point.” – **AN2F** |
| **Theme 2: Grain of salt with AI survival estimation** | |
| **Education to address lack of AI understanding** | **Limited view of AI.** In general, participants lacked a formal understanding of what AI is and how it works, often assuming all AI to be “like ChatGPT”. Though there were varying levels of comfort expressed by participants with some have done personal research into what AI, including taking a course or attending a conference (out of their own desire).  [In response to: What is AI?] “Yeah, ChatGPT. Just like a tool that can be used, a bit better than Google… So it’s like the new Google search.” – **RI14B**  “Because I’m also a researcher, I saw ChatGPT and the potential of AI and I was very curious about it. I also work in a department … with a very well-known researcher on AI applied to primary care. So I also had that exposure.” – **UZ11J** |
|  | **Misrepresentation of predictive AI as generative AI**. Participants commonly confused predictive and generative AI, with respect to how each works and the types of errors that can occur when using one vs. the other.  “I wouldn’t be comfortable using [it], because I am worried about hallucinations.” – **EE10S** |
|  | **Not comfortable explaining AI**. Participants described discomfort at the idea of justifying how AI operates to patients without being provided training on what AI is and how it works.  [In response to: How comfortable do you feel in defining AI to colleagues and patients?] “Not comfortable… I don’t really use it all that much. I haven’t read up on it too much. It’s mostly what I hear about it in passing, like in the news.” – **RI14B**  “I think telling someone ‘Hey – the AI looked at however many different factors and that’s the number it spat out’ is always going to be challenging for people to accept. I would certainly need a lot more training on how it comes up with what factors go into [AI] decision making and then how it decides on [a prognosis]. – **EE10S** |
|  | **Education focused on how it works and when to be cautious:** Participants describe needing to be taught what AI is, how to leverage AI, the patient data incorporated in prediction, how to interpret model performance (ex. accuracy and reliability), and indications/contraindications for use of AI. Participants emphasize that education around this needs to be provided to them prior to their use of AI, and that this information specific to each model performance and its should be communicated when models are deployed.  “I definitely need a lot more training on how [AI] comes up with the factors that go into its decisions … [I would need] general training on what AI is so [I] could explain it to patients, and they could understand how [it works].” – **EE10S**  **[**In response to: What would you need to know about AI?] “Starting from how people created these tools – it’s very important to know what the resources [used are]. What is the reliability of these tools? … How can we implement and use them in the best way. To what limit can we [predict]? How different is it [from human made results]? What are the precautions? We need real life examples.” – **ER7R**  **“[**AI education] should present a clear definition of the tool [in question] … how it was created in simple terms, how it was trained … practical implications like the accuracy of the tool, how it can be used, indications, contraindications … negative outcomes or the consequences of using it, the benefits.”  “This is more delivery, but case-based learning on the [clinical problem AI is hoping to address], sort of like a simulation. [It would be beneficial to] have somebody to talk to in case there are more questions as people start to use [the AI tool in question].” – **UZ11J** |
| **Concerns for new technology** **being wrong** | **Nobody wants to be wrong:** Participants note that AI’s prediction can be wrong or patient can be exceptions to general health trends. Participants caution the use of AI survival estimation for directing the care for these reasons. Participants expand, noting that this notion of not wanting to be wrong applies more generally with all prognoses in medicine.  “There’s no tool that’s necessarily super accurate, or you know, that’s going to be the gold standard, telling the truth all the time. And so with all tools, I think I would use this tool with a grain of salt, letting the patient know, ‘there’s this tool that’s quite accurate. But at the same time there is no tool, there’s no crystal ball that would tell the future […] to summarize, I think it would be a great tool, with some considerations for privacy and consent that that it would help guide discussions with patients, but also sort of used cautiously.” – **LS4A**  **“**It's just helping them understand that that’s the nature of the illness, and understand that wherever you are right now, in a functional point of view, they won't get better than this right now. […] And it's a difficult thing to get comfortable having those types of conversations with people and saying, I'm confident that you're not because nobody wants to be wrong, right? And I think sometimes we, even in medicine, we can think to some of those TV or movies, the media that we see where there's miracle recoveries from things.**” – OY3J** |
|  | **100% accuracy impossible to be a reality**: Participants support the potential clinical benefits that can be derived from AI survival estimation; however, they feel doubtful that a tool would ever be able to predict a timeline with 100% certainty. Specifically, residents note that AI cannot be accurate for everyone as some patients defy typical clinical trajectories. The certainty of AI models, and the representation of the patient in a models’ training data can help physicians to convey this information.  “I don’t feel like robots or machines can really work 100% accurately. I do believe in miracles. I do believe that things can happen that even doctors don’t believe is going to happen. […] So it will almost be like a study vs. a machine. We can only, you know, take what it says with a grain of salt.” – **IY1K**  “So, first of all, I would say, I think that I mean that's just never going to be possible in my mind. I think not that I'm an anti AI kind of guy, but I think AI learns everything from what we know, and I don't know that we know that for a lot of these patients it was possible. I mean, it's one of the things I always talk about with patients, too, is if you had a crystal ball, if you had the ability to predict, you would be a billionaire, because that's what everybody wants to know. And having that definitive, this is the timeline would definitely make those conversations easier. Like we said, if somebody who had heart failure somehow knew I'm gonna die in 2 weeks, I don't know what I'm gonna die of – if it's a heart failure, exacerbation, or I'm gonna get hit by a bus. But you know, if I know I'm gonna die, it's gonna change whether or not I want to come to the hospital. So, I think it would be super impactful to have that knowledge, but likely not possible to the degree that it would be effective.” – **OY3J** |
|  | **Concern for hallucinations in all AI tools**. Participants have concerns that the models can make up the answer; these concerns exist in additions to models being “off” or having a “margin of error,” though participants often confuse the type of AI where these outcomes are more likely.  “I'd probably be a little less comfortable [with an AI clinical decision support tool] just because there's so much more risk of like hallucinating and things like that. But yeah, if it was well validated. I understand that there's always the error bars on any sort of scoring system or whatever you're using. So yeah, I don't think it would change too much, but certainly be something in the in the back of my mind there.” – **EE10S** |
|  | **Transparency in model validation to create trust.** Participants reference the need to know and have evidence that the model has been validated and reflects the current Canadian guidelines. They describe increased trust when they can reference these guidelines.  “So I’d generally be very hesitant without that – really, that's why, going back to the very beginning, I want very strong validated data showing that this is accurate.” – **EE10S** |
| **Human clinical gestalt is important in prognostication** | **Human clinical gestalt is important in prognostication:** Participants describe that current approaches to prognostication involve features that cannot be quantified and that are based on clinician instincts and human factors.  “I think it’s always a gestalt thing when you’re looking at the patient… I think thinks like frailty, weight, work of breathing… It’s very gut feeling based most of the time.” – **EE10S** |
| **Need to ensure privacy with use of patient data** | **Privacy with use of patient data**: Participants emphasize concerns for patient privacy with the integration of an external tool generating predictions based on personalized patient data. Participants discuss the need to ensure patient consent prior to authorization of an external model to use patients’ data. Participants noted that this may change with social license that support to liberty in sharing data and comfort in privacy measures.  “I think it’d be a great tool, with some considerations for privacy and consent. It would help guide discussions with patients, but also sort of be used cautiously… Discussing with patients about this tool whether they want to use it and have their information be put into this tool” – **LS4A** |
| **Theme 3: Patient-driven use of AI for prognostication conversations** | |
| **Concerns for whether patients can handle accurate and specific prognosis** | **Not wanting to know exacts**: Participants note that with an AI survival estimation, there would still be variation in the degree to which patients wish to be informed and emphasize that patients would need to guide the use of an AI survival estimation for the management of their cases. Participants note that specific timing of mortality is of limited value to some patients with serious illness.  **“**I don’t know if I would like that, the exact week. Myself if I were in a situation where I had a terminal illness, I don’t think I’d want to know just because I don’t need to know the exact.**” – BD13A**  “I think it would change how I approach that conversation, because some people may not want to know, at least with that level of certainty.” – **AY5M**  “[I would communicate AI-drive prognosis] if the person wants to know. Because me as a physician, [will be] […] supporting the patient as much as they want, even though I know the date where the patient will die. I guess the question will be, if the patient wants to know, yes or no.”– **UZ11J** |
|  | **Potentially more harmful than good**: Participants have reservations about an AI survival estimation being beneficial in every patient situation. They describe concern that some patients would be harmed by the knowledge that they have limited time.  “In my mind, I just think it can do more harm than good, because you want the time that you have to be spending time with family and all. But in the back of your head, if there is like a timeline and there is like a ticking clock that you know it’s the end of the week or two weeks, whatever, it just ruins the whole experience, or it can really make it darker.” – **BD13A**  “But, again, with that being said, now I guess we go into the ethics of things where, it is ethical for me to be relaying this information? With the principles of non-maleficence and then beneficence, with this information am I doing more harm than good by replaying this information or not? I don’t know. I feel like kind of delves into a deeper topic there then.” – **AA18S** |
|  | **Physicians value specific prognosis, but do patients?** Participants note that specific prediction (e.g., anticipating, more accurately, the *exact* number of weeks a patient has) is of value to physicians, who can already estimate, in a broad sense, the time left. However, concerns exist around whether and how this can be communicated to patients. They discuss needing support around how to communicate this information and manage patients’ subsequent reactions.  “The question is … how is [AI predicted prognosis] any different than what we already do? Because nobody gives a specific number to a patient … it’s usually a ballpark … but it could be another piece of the puzzle that would make [the clinicians] even more confident” – **RI14B**  “I would probably talk to the patient about what they want to know – because obviously some people are not going to want that level of detail. [We would need to] have an opening discussion around, do you want this information, or is this something you might find more distressing than helpful.” – **EE10S**  “I think it would be very difficult to say: Here’s what the model says your exact date is … unless it really could take into account all of the different treatment options and palliative management type options” – **EE10S**  “I don’t think every patient would think it’s a good idea … it’s the emotional burden … many people will think no, I don’t want to know. I just want to live my days and think of the day ahead.” – **ER7R**  “I think the tool would be useful when the patient wants to know. But I’m sure not everybody wants to know how much time was left… I think informing patients is similar to conversations that we have with pregnant patients who want to get tested for a trisomy … I tell the patients, we have these tools, but we have to think – what would you do with the results? … The first consideration with these AI tools would be a conversation with patients: Do you want to know what the implications of knowing are?” – **UZ11J** |
| **Correct use case of patients who would benefit** | **Specifying target patient disease, and trajectory timeline**: Participants highlighted the need to clarify the utility of the AI tool with respect to the patient population, illnesses, and timepoint within the illness trajectory that the tool is applicable to, noting the variety of clinical applications and nuances in care of those with serious illness.  “And does this [accurate AI-predicted prognosis] refer to everybody? I can tell people who are 25 this or I can tell 80-year-olds this?” – **EN12S** |
|  | **Window of usefulness for survival estimation**: Participants note that patients and their family typically most value survival estimation near the end of life.  “But in terms of your more standard life-limiting illness, like the longer courses of cancer, I don't think it really changes that much because I'm coming from a place where there's that focus on the very early intervention on the palliative care side of things where we’re trying to get people two, three years out, so we’re having those conversations over time and adjusting appropriately. So, I think for those, it wouldn’t make a difference, but yeah, certainly, obviously, for sudden illnesses, sudden traumatic deaths and stuff like that, it would obviously change how we’d approach those patients.” – **EE10S** |
|  | **Ongoing conversation**: Participants note that care for patients with serious illness in family medicine is an ongoing conversation, with survival estimation only of value at certain points. Rather in such ongoing conversation, patients’ needs, and the emphases of the conversation vary over time.  “[Managing patients with serious illness,] I find it’s a prolonged conversation, it’s an ongoing conversation, based on how someone is doing at different checkpoints, but I think that the conversations are different each time, if that makes sense.” – **AA18S** |
| **Paternalism and Patient Autonomy** | **Patient autonomy for use of tool**: Participants emphasize the importance of considering patients’ perspectives on the degree to which they want to be informed alongside the utility impact of AI survival estimation.  “It’s tough because in some ways it’s respecting a patient’s autonomy versus beneficence […] if I offered that I had the information if they did want to know, and they really chose no, then I would say that I probably would not change that.” – **AU16D**  “If that does come up in the future, it has to be the patient’s choice if they wanted to know.” – **BD6A**  “It's something I would share. Maybe, if a study validated that this was really a good way to go then, yes, I would share that with my patient. But I would also say ‘this is what data says, but it's not something that always happens, right? That's the percentage. But take it as you will.’” – **IY1K** |
|  | **Confusion around paternalistic use of AI in patient care.** Misconception that with serious illness, the physician must decide whether the patient needs to know how much time is left (AI tool might want to have follow-up prompts for productive conversations).  [With current prognostication tools] “It’s not 100% accurate; it’s just an estimate based on what we see. So, it’s easy to couch in those terms of you’re not giving them the exact date and time type of thing. Whereas [with an AI tool], I think you would need to have the conversation ahead of time with your patient to make sure that it is really the information they want to have.” – **EE10S**  “I don’t know if I would tell the patient the specific number, but I’d probably give them a ballpark … I would use vague terminology. I wouldn’t give an actual specific number.” – **RI14B**  “Is it ethical for me to be relaying this information? Am I doing more harm than good by relaying this information or not?” – **AA18S** |
|  | **Consent discussions**: Participants emphasize their obligations to have consent discussions with patients before using an AI survival estimation. Participants note the need to pursue these conversations comes from hesitancy around whether patients want the information and from the novelty of AI (not yet accepted – in other words due to the lack of social licence for use without consent).  **“**I think one thing that would change right off the bat [with AI prognostication] would be a sort of a consent sort of issue. Discussing with patients about this tool whether they want to use it and have their information be put into this tool**” – LS4A**  “If the tool is accurate enough and has been approved for use in clinical contexts … I would probably let the patient know that the tool exists, the accuracy of the prediction, and have the conversation to inform the patient very well so that they could make a decision. Personally, I would not use it myself without considering the input of the patient… it would be the decision of the patient.” – **UZ11J** |
| **Patient-centered prediction outcomes of greater value than time left** | **Outcomes need to be usable by patients**: Participants emphasize that AI-prognostication would only be beneficial to patients if action could be taken from its predictions. They describe concern regarding providing information on predicted time of death if no actions could be taken to prevent the outcome.  “Many people would not want to know how much time is left and it could be devastating emotionally for the patients, if we can’t take control of the natures.” – **AN2F** |
|  | **Focus on current needs**: Participants describe that, as the patients’ physician, their role in each encounter depends on the patient’s needs in that specific visit – and these needs may or may not include survival estimation.  “I don’t care that she’s going to pass tomorrow, or in a month, or in a year. I need to cover her needs. […] we need to go day by day, I guess. The most important thing is what’s happening right now, not in the future, if that makes sense.” – **UZ11J** |
|  | **Predicting success more valuable**: Participants emphasize that the trajectory near end of life is similar for many patients and what is clinically more difficulty, and thus more valuable to have an AI tool for, is identifying the exceptions (e.g., medical miracles).  “But for the most part, I feel for conditions, we do have an idea as to what the trajectory of that is […]. So, regardless of if there was this server that could predict when you would die, knowing what conditions they have, I feel we already have an idea as to what the next few years are looking like for them. That being said, in terms of counselling, if I was aware that something had a great survival rate, but this patient was refusing any treatment, I think the best I can do is just to relay the facts that this is something that you likely will not die from.” – **AA18S** |
|  | **Prognosis is not everything:** Participants highlight that while prognosis is a part of care of serious illness, there is substantially more involved in the care of these patients including emotional support and connecting them to the necessary resources.  **“**The prognosis sometimes is certainly not everything and that there's more to that discussion and how you can support your patients than getting them ready with that information. There's a lot more that goes into the support of the patient and what how they make the decisions and things.**” – AY5M** |
|  | **Predicting symptom burden is more useful**: Participants describe that relative to the prediction of time alive left, predicting symptom burden is more useful with respect to end-of-life care among those with serious illness.  “[is there another predictive outcome that you think patients would value or want to know?] Yeah, I guess their odds of having palliative symptoms – like having severe pain in the future, severe constipation, shortness of bread – just so they are prepared to deal with this symptom that people have at the end of their life. If somebody probably is more likely to have pain, they are then very well prepared to me address it. Maybe even in advance, have some interventions, even psychological, to be prepared. Things like that.” – **UZ11J** |
| **Theme 4: AI augmenting, not replacing physicians** | |
| **Longitudinal therapeutic relationship is the core of family medicine** | **Caring, the art of medicine**: Participants describe leveraging trust and their longitudinal relationship with their patients to provide well-rounded support in the management of their serious illness.  “I’m providing holistic care, I’m taking in consideration every single aspect that could be related to the patient’s health, and I think this is the best approach that we can do, because there’s no definitive treatment for everything. There’s no best medical care approach. So, I think involving the patient in their care and letting them take the lead would help us also to make the best decisions.” – **ER7R**  “[When disclosing bad news] Would you like to sit down? Would you like us to call anyone, if you want someone else here with you, things like that? Asking them, how much do you want to even know about this? There’s bad news, but I will follow however you want this encounter to go. And they responded that they wanted to know everything, so that’s what we did. The way I like to do things, especially if I’m relaying lab results or imaging, I actually have a printout and then I go through line by line trying to explain a lot of it together.” – **BD13A** |
|  | **Salience of Therapeutic Relationship**: Participants describe the unique qualities of the relationship between the family physician and their patient. They describe such a longitudinal and trusting relationship as central to the management of serious illness.  “I think the best person to talk to their patients about their illness, their prognosis, is their family doctor because they would know the psychosocial aspect of the patient’s life, the patient’s expectations, the patient’s wishes, all that stuff. So, I think it is a crucial role. Very important role.” – **ER7R**  “As a family doctor, we are connected with the patients. We know their background, and they’ve been coming to us, and we have built that rapport and that trust, that relationship with them.” – **AN2F**  “They have a big role because they have the best relationship with the patient. They have a longitudinal relationship with the patient.” – **OY3J**  “It’s not only about trust, but it’s just even guidance or they’re looking to you for answers, building trust in that way. […] Rapport and trust are a big thing when it comes to patients dealing with serious illness because they look towards the healthcare provider for answers and for support. And there is so much uncertainty and questions, especially with certain illnesses, so I think that part of it is very important.” – **BD13A** |
|  | **Emotional Support**: Participants acknowledge their role, as family physicians, in providing emotional support to patients as part of the management of serious illness. They see themselves as the member of their care team that best understands their needs and opportunities for support.  “[Family doctors] They know them over multiple visits or years, are part of a supportive role with emotional support. They are involved with the care of the entire person, you know, whether they need more supports at home, home care, counselling […]. Family physicians may be in the optimal position to have these conversations. So, if we don’t have them, who is going to have them?” – **AY5M**  “In general, I would validate peoples’ emotions and validate how grief and sudden bad news, or dealing with a drastic life altering prognosis, like there are a lot of feelings that come up and whatever feelings they have are okay. And it’s okay to take the time to process things. Mostly just asking patients what we can do to help them and what they would like. A lot of times that looks like getting a patient navigator like a social worker, or spiritual navigator, involved.” – **AS17M** |
|  | **AI can re-direct time and focus to the therapeutic relationship:** Participants not that AI would not replace the physician, but rather redirect time and energy towards other elements of the therapeutic relationship including eye contact, emotional support, etc. Indeed, accurate prediction of outcomes of the collection of patient data to predict these outcomes, is only a small role of the therapeutic relationship.  [Referencing a colleague that uses an AI-based scribe tool in their practice] “He says that he has time to talk to the patient face to face, which is a positive … [Especially in conversations around end of life] it would be better if you could be face to face with patients and not writing down the note”. – **UZ11J**  “I don’t think [AI] can replace a doctor, but I think it will make the next generation of doctors far more efficient than non-AI physicians.” – **RI14B**  “Honestly, it’s great – especially in terms of time saving.” – **ER7R**  “[AI] improves patient centered care, because it can take something off your shoulders, like documenting, typing, paperwork… it will take less time and you save more time for the patient.” – **ER7R** |
| **Physicians with AI will replace those without AI** | **Filling in the gaps**: Participants emphasize that a major role of family physicians is addressing the gaps in care including providing information, priming patients for visits with specialists, coordinating care to specialists as well as community resources.  “Are their goals of care to stay at home or do they have plans to go to LTC? What are their supports like in terms of family members? The social side of things. Managing medication side-effects more so than the actual medications. Things like that, just try and fill in the gaps around the specialist’s care. And then, I think the other benefit, because we have some extra time sometimes in clinic to have these bigger goals of care type discussions as well and make sure everyone is on the same page” – **EE10S** |
|  | **Won’t change my practice:** Participants describe that even knowing the prognosis more accurately, their roles as a family physician would not change; they would continue to support the patient to the extent they desire.  “Because me as a physician, I can know [the AI prognosis] and I don’t think that will change my practice. I want to be there for the patient, covering all their needs or supporting the patient as much as they want, even though I know the date where the patient will die.” – **UZ11J** |
| **Efficiency looks different physician-to-physicians** | **I can also do it accurately**: Participants note that prognostication of serious illness is a skill they are also able to do and do so accurately.  “I think even if we used AI, and even if it was shown that it was highly predictive, it can’t tell the future. So, maybe AI just speeds up the information gathering. But I can also tell a patient how many weeks that they probably have left, and in some cases, it could be very accurate, in some cases it might not be so accurate.” – **RI14BB** |
|  | **Credible sources needed with automation.** Participants value the efficiency of AI and feel comfortable automating tasks within their practice when credible sources are referenced in AI output.  “[With current use of an AI tool in clinic] You type your question and then you get a very straightforward answer. In case I would be concerned about the service, I know that [the AI tool] was trained with the Canadian guidelines. And then I also can click on the references because each response has references. So that makes me more comfortable… As I said, I use it to speed things up and get specific, evidence-based answers” – **UZ11J** |
|  | **Efficiency gain is task and user dependent**. Participants describe specific areas within their practice where AI has previously increased efficiency and describe the clinical situations in which an AI tool may be used to augment their workflow. Participants note that all physicians will not experience the same gains in efficiency from each AI tool, noting that AI tools **(**e.g., AI scribes) may increase the efficiency for one physician while slowing down another.  “There's this thing called Hippo AI! It's like, it's like ChatGPT for doctors, and I'm on the beta. I'm using it as they're kind of testing it out. So, they gave me access to it. and sometimes I'll use it in clinic – like I'll look up stuff. I'll look up guidelines. Sometimes I'll look up like sort of general stuff. I found it helpful so far, but I don't use it regularly enough. […] It depends on what I'm asking. This one in particular will send me references, if it gives me a recommendation. It'll tell me like where which guideline it pulled it from. So, I’m a little bit more confident that, and say, just generic like ChatGPT. But then for Google, sometimes, that's a little bit better for me, because I know exactly it. That's for like when I know exactly what I'm looking for – like a hypertension guideline or something like that, like, I know exactly what I need. Then Google, sometimes a little bit faster. But if it's something kind of like broad and more like if I'm asking it to do something a bit more complex. And yeah, definitely. Hippo AI is pretty useful.” – **RI14B** |
|  | **Efficiency looks different physician-to-physicians**.  **“**Our clinic just rolled out an AI scribe where you just press a button. It listens to the conversation. It just makes a note. And it's this incredible thing for the old school doctors who can't type and talk at the same time. It's a godsend. I tried it. I didn't really like it. I felt the note that it generated was very clunky and didn't look very good, not very organized. I liked my way of doing it because I use a template. And then I just type while I talk and fill out the template. But I can see how somebody who cannot type and talk, who has to draft up a note after the interaction is gone and patients out the door, how an AI would be really good. Super helpful.” – **RI14B** |
| **Physicians to guide introduction of AI in clinical care** | **Physicians AI leaders**. Participants reference the salience of physician leaders in AI implementation so that there is consideration to the patient perspective and trainee perspective, alongside that of institutional policy makers and established clinicians. This includes the consideration towards the AI use in the medical training of residents.  “I work in a department at [university name] where there is a very well-known researcher on AI applied to primary care. I had that exposure… she was giving talks and stuff, so I was also very interested.” – **UZ11J**  [In response to what types of learning experiences and exposures are necessary for clinicians to confidently implement AI into their practice] “I think they just have to be given free access to it… People should be encouraged to use it and to experiment with it. Veteran users should demonstrate how good it is and encourage new learners to try it out. There should be as few barriers as possible. It should be made easy for new docs to try it out.” – **RI14B** |
|  | **Practical experience using AI:** Participants emphasize that, as a new technology, education on AI and practice with AI tools is needed outside of the clinical setting to create comfort and confidence. Participants describe that such experiential learning could take shape of a simulation; this format is already used for learning how to have conversations around serious illness.  **“**Also – this will be more delivery – but like case-based learning on the problem, problem-based learning, some sort of like simulation. And then having somebody to talk to in case there are more questions as people start to use it.**” – UZ11J** |
|  | **Reduced barriers to using AI:** Participants emphasize that to facilitate adoption of AI – specifically with technology that is so revolutionizing and potentially altering of care pathways – access needs to be as simple as possible to encourage its use. Participants commonly referenced ChatGPT, as an example whether the technology was so easily (and cheaply) available for people to determine how it works and how each can individually leverage it.  “[Regarding the type of exposure needed to access it,] I think they just have to be given like free access to it. It has to be readily available. you know, don't charge people. You should make it very easily accessible. People should be encouraged to use it and to experiment with it […] Basically, there should be as few barriers as possible. It should just be made easy for new docs to use to try out” – **RI14B** |
|  | **Modeling of when and how to use it:** Participants discuss that with current AI technologies, they look to peers and supervisors on how and when they use AI in their care of patients, noting which patients, patient encounters, illnesses, etc. the tool best applies to.  “I saw my supervisor using [an AI scribe tool] and he was encouraging me to use it. [My supervisor] says that he has more time to talk to patients face. That’s a positive, for sure.” – **UZ11J** |
