# Supplementary material for: Perspectives of family medicine residents on artificial intelligence for survival estimation in patients with serious illness
Source: PLOS Digit Health. 2025 Jul 1;4(7):e0000917. doi: 10.1371/journal.pdig.0000917 (PMC12212547; doi:10.1371/journal.pdig.0000917)
Supplement: S5 File — (DOCX) [file pdig.0000917.s005.docx]

**Study Protocol: Perspectives of Family Medicine Residents on Artificial Intelligence for Survival Estimation in Patients with Serious Illness**

# *Below is an abridge protocol submitted to our Research and Ethics Board.*

# OVerview of STUDY METHODS

The study will leverage qualitative methodology, conducting and analyzing semi-structured interview data from 18 family medicine residents (i.e., post-graduate medical trainees specializing in family medicines) in Canada. In doing so, we will employ grounded theory, a research approach that uses qualitative and quantitative data to develop insights into the social processes that shape work and experience.[1–3] Our approach to grounded theory draws from constructivist grounded theory.[4,5]

We will collect interview-based data from Canadian medical learners. Recruitment will begin from a convenience sample of family medicine residents at the University of Toronto; then, as needed, we will recruitment expand geographically and disciplinarily to include additional postgraduate programs and institutions. We will also use a process called ‘nominated sampling’ where current participants nominate peers targeted to inclusion criteria.[6] All interviews will be conducted online using an internet platform such as Zoom. Human subject data collection in this study will include the audio of formal semi-structured interviews. Only the audio of the online meeting will be recorded. The audio recordings will encrypt and password protected. Interview recordings will be transcribed either by a member of the research team or by a professional transcription service hired through the Department of Family & Community Medicine and transferred using a secure encrypted server as per departmental protocol. The formal interviews are used to directly discuss analytical concepts that are emerging in the work of constant comparative analysis in relation to the participant’s work experience. Based on the initial data collected, the research team may contact participants and invite them to participate in a follow-up interview that includes the letter of information and consent. Interview recordings will be transcribed and stored using the same approach.

This study follows an emergent design. As such, the initial interview guide will not be the final interview guide. However, it is possible to be assured that once the interview guide is generated from the initial questions and evolves throughout the research process it will remain focused on the research question at hand.

For data analysis, theoretical coding will identify emergent themes in the interview data. When the process of theoretical sampling has allowed for sufficiently meaningful theoretical findings—a state called ‘sufficiency’ by grounded theorists —the collection of new data ends and the process of manuscript writing takes precedence.[7] Due to the breadth of the research questions and previous grounded theory studies in health care examining similarly broad questions that have collected data from large populations of participants, we have allowed that the data collection process may require a large number of interviews (i.e. maximum of 200)[8] before sufficiency is reached.

# STUDY PARTICIPANTS

## participants

All medical learners specializing in family medicine (e.g., medical resident physicians) are eligible to participate. There are two exclusion criteria all participants cannot be under 18 years of age and must be able to read and communicate in English.

Some personally identifiable information will be collected from each participant. For medical learners, this will include name, email addresses, program of training (if applicable), and year of training (if applicable) to coordinate participation. For patients, this will include name and email address. Before beginning their participation, participants will be asked to sign a consent form that will allow the researchers to collect this data. As discussed in *Section 11 – Confidentiality and Privacy*, this personal information will be separated from the study data and anonymized using a unique code for each participant.

## Recruitment

Recruitment will occur in four phases. In the first and second phases, we will recruit through email and in person from a convenience sample of resident physicians and their preceptors at the University of Toronto. Initially, a recruitment email will be sent from the administrator of residency programs and sites that agree to participate. A follow up email will be sent four to eight weeks later. Following initial insights from the data collection process, recruitment will expand geographically and disciplinarily to include additional postgraduate programs and institutions. We will also use a process called ‘nominated sampling’ where current participants nominate peers targeted to inclusion criteria. In person, we will request an opportunity to recruit through an oral presentation to residency programs during professional development days and grand rounds.

## Compensation

At this time, participants will either be compensated with a small honorarium ($50-100) for participating or not be compensated for their time due to a lack of resources. We do not believe that the burden of participation is high enough for participants (interviews will last approximately 30-60 mins) to make a lack of compensation inappropriate. The Letter of Information (LOI) sent to participants will discuss compensation if available (both versions of the LOI & Consent have been included in this protocol).

# Risks and Benefits to Participation

## Psychological/Emotional Risk

There are minimal risks associated with participating in this research. Discussions of serious illness are within the scope of routine patient-clinician interactions. The opportunity to debrief any adverse events that occur during the interviews will be made available to participants if needed.

Virtual communications platforms (such as Zoom) are inherently vulnerable to compromises in privacy. However, all interviews will be anonymized, and entry will require a passcode for participation.

Discussions of serious illness are within the scope of routine patient-clinician interactions. However, it is possible that, for some participants, discussions around death and dying may result in emotional distress, anxiety, or worry. Should they experience these concerns, supports are available to each participant through their respective wellness support bodies. For medical residents, the contact sheet for local support resources for residents at each medical school nationally is available through the [Resident Doctors of Canada crisis resource](https://residentdoctors.ca/resources/crisis-resources/)s. This information will be provided to participants within the letter of information.

## Benefits to Participating

Improved understanding of advance care planning will improve teaching around patients with complex, life-limiting illness in medical education. In time, this study aims to help patients with serious illness receive medical care that is in keeping with their values.

# Informed Consent

## Consent

Prospective participants will receive a recruitment email offering them the opportunity to participate in the study. This email will include the LOI and consent form for the study. If prospective participants are interested in participating, they will be able to read the LOI on their own time and, if they do not have questions, sign the consent form. If the prospective participants have further questioned the LOI will detail an email address to use in order to clarify any issues before signing the consent form. Once the consent form is signed the participant will email a copy of the signed consent to the researchers (as detailed on the consent form).

Consent forms will be sent and received only from encrypted institutional email addresses of the principal investigator or assigned delegate. Once signed, they will be stored separately from the master list (see Section 10) on encrypted and password protected institutional servers.

## Withdrawal of Consent

The letter of information for interviewees will include the following on withdrawal from the study:

*If you volunteer to be in this study, you may withdraw up to two weeks after participation. Withdrawal will in no way affect your professional role. You may indicate to the researchers via email your desire to withdraw. You may also refuse to answer any questions you don’t want to answer and still remain in the study.*

If a participant chooses to withdraw from the study, their data will be removed from the study – the interview recordings, transcripts, and any notes taken will be permanently destroyed. Withdrawal from the study will have no effect on the participant.

After two weeks, the data collected will be incorporated into the dataset and will be coded using thematic codes. After themes are assigned, the nature of the dataset is permanently changed, and data can no longer be functionally withdrawn.

# PArticipant CONFIDENTIALITY

At the beginning of the study, each participant will be assigned a codename (e.g., P237). After transcription of the interview recordings, any identifying information will be removed and replaced with the codename. A single master list containing the name of participants and their codenames will be created in order to link demographic information with the anonymized interview data. This master list will be stored in a password protected document on the password protected personal computers of the principal investigator (TA).

The interview recordings will be encrypted, password-protected, and stored on the password protected personal computer of the principal investigator. The recordings will be transferred to a professional transcription service using a secure encrypted server as per departmental protocol. This second version of the recording will be destroyed after transcription. The de-identified transcripts will be transferred back to the research team using a secure encrypted server. All de-identified data will be stored on the password protected personal computers of the researchers.

De-identified data will be stored for seven years before it is permanently deleted. The audio recordings, master list of names, year of training, and program with unique assigned codenames will be permanently deleted after seven years.

# Planned Data Analysis

## Data Analysis

We will use a pragmatic framework to conduct our analysis, drawing on the principles of constructivist grounded theory[5] and inductively analyzing all transcripts.[9–11] Our approach will recognize and transparently note our positionality as authors and the theory that underpins our methodological approach. Meeting notes will be recorded to establish an audit trail.[1]

Data will be coded to highlight insights and patterns in participants’ perspectives. Coding will be done in duplicate with *NVivo* software (version 14)[12] after initial thought calibration following completion of the first five transcripts. Authors will compare coded transcripts for consistency in coding, discussing with the larger research groups where discrepancies exist. Development of the initial codebook will be done iteratively by the coding authors; this will occur following coding and take place through collaborative working sessions, in which their codes will be triangulated to reach a consensus structure. Where possible, *NVivo* labels were used to retain participants’ original thoughts.[5]

Validation of the final codebook will involve the larger research team and consist of peer feedback through reviewing coded transcripts and discussing the alignment of the themes with associated codes. Disagreements about codes or themes will also be resolved through consensus discussions.

## Data Reporting

In reporting our findings as a manuscript for peer reviewed publication, we will report the parent codes with an exemplary quote in textboxes to remain concise. We will present the full codebook as an appendix for transparency. All quotes will be presented with a unique and randomly generated study participant identification code to maintain confidentiality of participants but enable readers to evaluate differences within and between participants. Reporting will follow the Standards for Reporting Qualitative Research guidelines.[13]

# References

1. Glaser BG, Strauss AL. The discovery of grounded theory: strategies for qualitative research. 5. paperback print. New Brunswick: Aldine Transaction; 2010. ISBN:978-0-202-30260-7

2. Fakis A, Hilliam R, Stoneley H, Townend M. Quantitative Analysis of Qualitative Information From Interviews: A Systematic Literature Review. Journal of Mixed Methods Research 2014 Apr;8(2):139–161. doi: 10.1177/1558689813495111

3. Creamer EG. Advancing Grounded Theory with Mixed Methods. 1st ed. London: Routledge; 2021. doi: 10.4324/9780429057007ISBN:978-0-429-05700-7

4. Apramian T, Cristancho S, Watling C, Lingard L. (Re)Grounding grounded theory: a close reading of theory in four schools. Qualitative Research 2017 Aug;17(4):359–376. doi: 10.1177/1468794116672914

5. Charmaz K. Constructing grounded theory. London ; Thousand Oaks, Calif: Sage Publications; 2006. ISBN:978-0-7619-7352-2

6. Trotter RT, Needle RH, Goosby E, Bates C, Singer M. A Methodological Model for Rapid Assessment, Response, and Evaluation: The RARE Program in Public Health. Field Methods 2001 May;13(2):137–159. doi: 10.1177/1525822X0101300202

7. LaDonna KA, Artino AR, Balmer DF. Beyond the Guise of Saturation: Rigor and Qualitative Interview Data. Journal of Graduate Medical Education 2021 Oct 1;13(5):607–611. doi: 10.4300/JGME-D-21-00752.1

8. Mottram A. ‘They are marvellous with you whilst you are in but the aftercare is rubbish’: a grounded theory study of patients’ and their carers’ experiences after discharge following day surgery. Journal of Clinical Nursing 2011 Nov;20(21–22):3143–3151. doi: 10.1111/j.1365-2702.2011.03763.x

9. Morgan D. Pragmatism as a Basis for Grounded Theory. TQR 2020 Jan 13; doi: 10.46743/2160-3715/2020.3993

10. Mohajan D, Mohajan HK. Constructivist Grounded Theory: A New Research Approach in Social Science. RAE 2022 Oct;1(4):8–16. doi: 10.56397/RAE.2022.10.02

11. Rieger KL. Discriminating among grounded theory approaches. Nursing Inquiry 2019 Jan;26(1):e12261. doi: 10.1111/nin.12261

12. Lumivero. NVivo (Version 14). Denver, Colorado; 2023. Available from: www.lumivero.com

13. O’Brien BC, Harris IB, Beckman TJ, Reed DA, Cook DA. Standards for Reporting Qualitative Research: A Synthesis of Recommendations. Academic Medicine 2014 Sep;89(9):1245–1251. doi: 10.1097/ACM.0000000000000388
